# Protein-bound nitrosyl iron complexes: long-lived physiological form of nitric oxide

**DOI:** 10.4103/mgr.MEDGASRES-D-25-00094

**Published:** 2026-01-06

**Authors:** Olga V. Kosmachevskaya, Alexey F. Topunov

**Affiliations:** Bach Institute of Biochemistry, Research Center of Biotechnology, Russian Academy of Sciences, Moscow, Russia

Nitric oxide (NO^•^) is a gaseous molecule poorly soluble in water. It has an unpaired electron on the nitrogen atom, which gives the molecule paramagnetic properties. Along with other small gaseous molecules (O_2_, CO, and H_2_S), NO^•^ belongs to the group of gas transmitters.

NO^•^ reacts readily with various substances, yet its lifetime is extremely short, and the molecule itself is not capable of traveling long distances. It was therefore clear that for proper functioning and transportation in the organism over a relatively long time, NO^•^ had to be included in some donor substances. Back in 1980, future Nobel Prize winner Louis Ignarro, with his co-worker Carl Gruetter, showed that NO^•^ metabolism was the underlying mechanism behind the vasodilatory action of long-known drug glyceryl trinitrate (nitroglycerin).^1^ Subsequently, numerous studies were carried out in the fields of pharmacology, biochemistry, and physiology. The former aimed to find out some promising NO^•^ donors to be potentially used as a medicine, while the latter set out to elucidate natural physiological NO^•^ metabolites and the mechanisms of their action.

**Nitrosyl iron complexes – a system for NO^•^ storage and processing:** The organism itself has well-developed systems to stabilize and deliver NO^•^ to biological targets. They include nitrosothiols (R-SNO) and nitrosyl iron complexes, most often, dinitrosyl iron complexes (DNICs). DNICs are some of the main forms of NO^•^ storage in the organism, and incorporating NO^•^ into these complexes not only enables its transport, but also stabilizes the molecule itself, preventing it from being rapidly destroyed in reactions with free radicals. The main functional fragment of DNICs is an iron-dinitrosyl one [Fe(NO)_2_].

Low-molecular-weight DNICs in the aqueous solution are present in two forms: mononuclear (M-DNICs) and binuclear (B-DNICs), and both exist in nature. In M-DNICs bivalent iron coordinates two NO^•^ molecules and two anionic ligands, which are most often thiol or imidazole groups of cysteine and histidine residues, respectively. The structure of B-DNICs is still being debated. Some researchers believe that sulfur atoms bind iron in these complexes in the same way as in Roussin’s red salt: K_2_[Fe_2_S_2_(NO)_4_]. According to another, more popular opinion, B-DNICs are dimeric associates of mononuclear complexes (**Figure 1**). The equilibrium between M-DNICs and B-DNICs depends on the pH and concentration of thiols (RSH):

**Figure 1:**
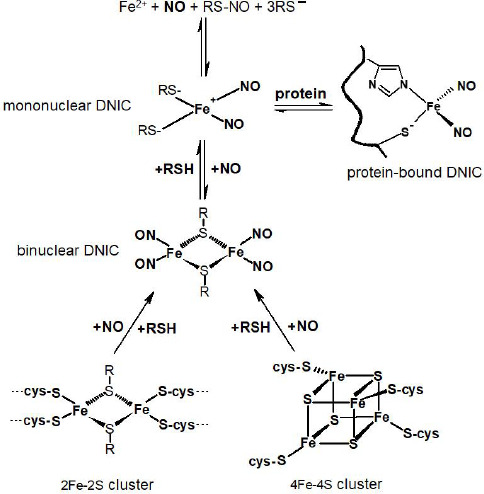
Transition of mononuclear, binuclear, and protein-bound DNICs. DNIC: Dinitrosyl iron complexes; RSH: *L*-cysteine, glutathione, and other thiols; RS-NO: S-nitrosothiols.

[Fe^+^_2_(µRS)_2_(NO)_4_] + 2RSH ←→ 2[Fe^+^(NO)_2_(RS)_2_]^–^ + 2H^+^.

DNICs can transfer from low-molecular-weight ligands to high-molecular-weight ones and vice versa. Cysteine and histidine residues can also be a part of high-molecular-weight compounds: peptides and proteins, mainly albumin and hemoglobin (Hb). The ligand structure determines the stability of DNICs, which increases in the following order:

Cysteine DNICs (Cys-DNICs) → Glutathione DNICs (GSH-DNICs) → Protein-bound DNICs.

DNICs with low-molecular-weight ligands are the most studied, although protein-bound DNICs are stable and physiological. Even in a recent review describing DNICs as a “working form” of NO^•^,^2^ insufficient attention has been paid to the protein-bound DNICs.

Due to the paramagnetic properties of NO^•^, electron paramagnetic resonance (EPR) spectroscopy is one of the most important ways to gain valuable insights into NO^•^. Unfortunately, there are no straightforward methods for detecting the protein-bound DNICs, though the abovementioned EPR spectroscopy is commonly used to detect and quantify them. This method was used in the first detection of protein-bound DNICs. They were discovered back in the 1960s by the EPR signal with a g-factor of 2.03.^2^ Since NO^•^ is synthesized in different cells, DNICs have been found in both prokaryotic and eukaryotic organisms under nitrosative stress, excessive formation of reactive nitrogen species. The paramagnetic centers localized on DNICs with two thiol-containing ligands [(RS^–^)_2_Fe(NO)_2_] are responsible for the signal with the g-factor 2.03. Such complexes are formed on proteins containing iron-sulfur clusters nitrosylated by NO^•^, such as proteins involved in electron transport chains and redox-sensitive transcription factors.

Despite its widespread use, EPR spectroscopy has its limitations ― it allows for detecting only the mononuclear paramagnetic complexes. Meanwhile, Mukosera et al.^3^ proposed the ozone-based chemiluminescence method, capable of detecting both mononuclear and binuclear complexes. The protein-bound DNICs could also be detected via nuclear resonance vibrational spectroscopy.^4^ Since the concentration of B-DNICs is difficult to determine, it is likely that the total DNICs content in cells is significantly higher than the usually measured one (0.5–0.9 nmol/mg protein).

**Properties and physiological role of nitrosyl iron complexes:** The therapeutic value of NO^•^ is achieved through nitrosylation of iron in heme groups and iron-sulfur clusters, which represent the prosthetic group of many proteins. The nitrosylation of the heme iron in soluble guanylate cyclase leads to vascular relaxation and thus decreases blood pressure. The role of NO^•^ goes far beyond regulating blood vessel tone and is crucial for many systems, including neural signaling and protection against pathogens.

Nitrosylated iron-sulfur clusters served as a model for designing biomimetic complexes, described by the general chemical formula: [Fe_2_(SR)_2_(NO)_4_], where R stands for aliphatic or aromatic thioamines (penicylamine, cysteamine, and aminothiophenol). The work on synthesis and studying these complexes is carried out in the Institute of Problems of Chemical Physics (Chernogolovka, Moscow region, Russia), which has a library of complexes with varying stability, NO-donor activity, and the ability to penetrate cells. The toxic effect of the synthesized anionic DNICs on some tumor cells, for example, glioblastoma, has been reported.^5^ DNICs modified with various thiolate bridging ligands showed a high cytotoxicity against human cervical cancer cells.^6^ These complexes could be coordinated with Cys102 residue of ferritin that enhanced NO^•^ cytotoxicity. Nitrosyl iron complexes may selectively suppress tumor development, which is based on rapid destruction of complexes in the actively dividing cells and a subsequent release of large amounts of NO^•^.

The organism undergoes a rapid transition of the ferro-dinitrosyl fragment from low-molecular-weight ligands to the protein ones, with further slow decomposition and release of NO^•^. More often than not, DNICs are detected in blood, where they are bound with serum albumin and erythrocytic Hb. It has been shown that DNICs bound with blood proteins are formed in rabbit erythrocytes upon intravenous injection of DNICs with thiosulfate.^7^ Nitrosyl iron complexes can bind to a protein in various ways, forming a coordination bond and adsorbing on its surface due to weak intermolecular interactions.^8^

Since nitrosyl iron complexes are a natural way of NO^•^ binding, they can be used as an effective and safe medicine, NO^•^ donors. Their effect in animal experimental models is being studied for regulating blood pressure, protecting the myocardium in ischemia, hypertension, stroke, treatment of pulmonary hypertension, cancer, as well as for healing skin wounds.^2^ Complexes with natural thiol ligands (DNIC-RS), glutathione, cysteine, the so-called endogenous DNICs, can be synthesized most easily in the laboratory, and this process can be potentially transferred to the semi-industrial and industrial levels.

Besides regulatory and therapeutic effects, DNICs and products of their decomposition can also display pro-oxidant properties. The iron (Fe^2+^) released from the complexes can catalyze peroxidation process in the Fenton reaction:

(Fe^2+^ + H_2_O_2_ → Fe^3+^ + OH^•^ + OH^–^).

NO^•^ can lead to the formation of peroxynitrite in a diffusely-controlled reaction with superoxide. In addition, DNICs destroy iron-sulfur clusters of proteins, including the ones involved in cell respiration. Therefore, studying their antioxidant/prooxidant effects in various experimental systems is necessary for DNIC implementation to medicinal practice.

Our studies show that DNICs of various types exhibit primarily the antioxidant properties in the model systems.^9^,^10^ We further proposed the cytoprotective mechanisms and antioxidant activity of DNICs. An important role of the complexes is to neutralize the prooxidant effect of “free” iron, which leaves intracellular depots during oxidative stress. The fact that the reaction rate of albumin-bound DNICs with superoxide (k ≍ 10^7^ M^-1^s^-1^) is almost three times lower than the rate of the superoxide interaction with NO^•^ (k ≍ 6.7 × 10^9^ M^-1^s^-1^) proves that DNICs stabilize NO^•^. The balance between antioxidant/prooxidant properties of nitrosyl iron complexes depends on their chemical and electronic structure, concentration, and the rate of NO^•^ release.

**Therapeutic potential:** Intracellular DNICs are believed to be mainly represented by protein-bound forms. Many protein-bound DNICs are formed by ligand exchange in reactions between low-molecular-weight ones and proteins. The balance between these two DNIC types is determined by the content of cysteine and glutathione, and a decrease in the pool of which shifts this balance towards more stable protein-bound DNICs (**Figure 1**).

In the organism, DNICs with thiol ligands are formed from iron-sulfur proteins or are drug-delivered. By binding to DNICs through its low-molecular-weight analogs, albumin (or another protein) promotes NO^•^ delivery to specific tissues and cells. It is assumed that DNICs in animal tissues present predominantly in binuclear form, which is much more stable than mononuclear one. There is an equilibrium between different forms of complexes in the organism (**Figure 1**), which allows them to gradually release NO^•^ as its concentration in cells decreases. This provides for a more stable and prolonged effect, unlike other donors that can cause sudden spikes in NO^•^ concentration.

Low-molecular-weight DNICs form stable protein-bound DNICs in blood and organ tissues, which ensures the gradual release of NO^•^ as its concentration decreases. This results in a long-lasting hypotensive effect in humans. This is the main advantage of thiol-containing DNICs compared to other NO^•^ donors. Albumin-bound DNICs are a potential pharmacological drug that can be used for NO^•^ delivery in antioxidant and anti-inflammatory therapy. Further research is needed to fully understand their mechanisms of action and potential therapeutic applications.

The binding of DNICs to protein amino acid residues can affect the structural and functional properties of the proteins themselves. Incorporating thiols of cysteine residues in DNICs composition can modulate thiols reactivity.^11^ If DNICs are formed on cysteine-based redox-switch proteins, complexes modulate the susceptibility of these proteins to changes in redox conditions. In addition, DNICs can serve as site-specific antioxidants protecting reactive protein thiols against oxidation.^9^

In addition to thiol-containing ligands, imidazole of histidine may be included in DNICs. Such complexes are formed on the dipeptide carnosine (β-alanyl-*L*-histidine).^10^ Carnosine-bound DNICs were obtained for the first time in our laboratory in Bach Institute of Biochemistry. They can combine the therapeutic potential of both components: NO^•^ and carnosine, and are promising for developing the pharmacological drugs aimed to treat cardiovascular and cancer diseases, type 2 diabetes mellitus, and neurodegenerative disorders. We have also shown that DNICs can be formed on the histidine of albumin protein.^10^

Water-soluble DNICs [Fe_2_(µ-SCH_2_CH_2_OH)_2_(NO)_4_] are also being studied for oral NO^•^ delivery to the brain for activating the hippocampal neurogenesis.^12^ Such delivery is carried out by endogenous conjugation with gastrointestinal glycoprotein mucin and serum albumin with a controlled NO^•^ release under fine-tuning of the medium pH or at the illumination with light of a certain wavelength.

The inhaled NO^•^ has been used in medicine for as long as 30 years to improve the coronary blood flow and to treat pulmonary hypertension in newborns and adults. Inhaled DNICs injection can be used to treat lung diseases, such as pulmonary hypertension, as well as inflammatory processes, including inflicted by viruses. For example, some thiol-containing DNICs release NO^+^, which is responsible for their cytotoxic and antiviral (e.g., anti-severe acute respiratory syndrome coronavirus 2) effects.^2^ Initially, NO^•^ from gas cylinders was applied for this purpose. However, it is unsafe to use NO^•^ in this way, as it is an unstable gas that can turn into harmful compounds during storage. Currently, portable NO^•^ generators are used for inhalation therapy, which produce it directly next to the patient, minimizing the formation of toxic by-products. The use of stabilized NO^•^ forms, which include nitrosyl iron complexes, existing in the form of freeze-dried powder or crystals, prevents many of the problems associated with gaseous NO^•^ delivery systems.

Various NO^•^ donors are used for treating cardiovascular pathologies. A special group of such donors are organic nitrates (nitroglycerin, isosorbide dinitrate, and isosorbide mononitrate). After long-term use of nitrates, approximately 70% of patients received tolerance. The group of thiol NO^•^ donors also include low-molecular-weight S-nitrosothiols (R-SNO), which are not used in treatment due to their potential toxicity and lack of clinical verification. Many nitroso compounds display a strong carcinogenic effect as well. S-nitrosoglutathione can cause nitrosative stress by generating reactive nitrogen species that can damage DNA. This was also shown in our studies.

Unlike nitrates, which have an obvious short-term effect, thiol DNICs have a long-lasting hypotensive effect grounded in the mechanism of stable protein-bound DNICs being formed. In addition, DNICs do not cause tolerance, and they exhibit an evident therapeutic effect at lower concentrations.

**Conclusions and future perspectives:** In biomedicine, DNICs with low-molecular-weight ligands are usually applied. Protein-bound DNICs have not been used as medicinal substances yet, although they are more physiological. It is the protein DNICs that are the endogenous form of long-lived NO^•^. DNICs associated with albumin and Hb play a critical role in the conservation and transport of NO^•^ in human and animal circulatory system.^7^,^8^ These protein-bound DNICs also serve as reliable biomarkers of oxidative stress, as they are destroyed in the presence of superoxide.

Stabilized NO^•^ preparations can be used in soft medicinal forms, such as ointments, creams, gels, and suppositories, which are intended for local use without affecting the systemic blood flow. Carnosine DNICs can also be used in medicinal cosmetics, since both NO^•^ and carnosine have dermatoprotective properties. If freeze-dried in an oxygen-free ampoule, protein-bound DNICs are also quite stable. It can be assumed that albumin-bound DNICs may be used for treating various skin diseases. Regardless of the method of NO^•^ injection, its binding to proteins in the form of nitrosyl iron complexes not only makes it possible to stabilize and store this gas transmitter ― NO, but also reduces its cytotoxicity.

Protein-bound DNICs are endogenous long-lived NO^•^ intermediates, without which NO^•^ functioning in the organism is highly hindered. Therefore, it is becoming increasingly important to study the metabolic pathways of the protein-bound DNICs, which will prospectively open new vistas for a better understanding of features of NO^•^ functioning as a signaling and regulatory molecule with a remarkable therapeutic potential.

## References

[1] Ignarro LJ, Gruetter CA (1980). Requirement of thiols for activation of coronary arterial guanylate cyclase by glyceryl trinitrate and sodium nitrite: possible involvement of S-nitrosothiols. Biochim Biophys Acta.

[2] Vanin AF (2024). Mechanisms of the formation and function of dinitrosyl iron complexes as a “working form” of nitric oxide in living organisms. Biophysics.

[3] Mukosera GT, Liu T, Ahmed ASI (2018). Detection of dinitrosyl iron complexes by ozone-based chemiluminescence. Nitric Oxide.

[4] Tonzetich ZJ, Wang H, Mitra D (2010). Identification of protein-bound dinitrosyl iron complexes by nuclear resonance vibrational spectroscopy. J Am Chem Soc.

[5] Sanina NA, Kozub GI, Kondrat’eva TA (2022). Anionic dinitrosyl iron complexes – new nitric oxide donors with selective toxicity to human glioblastoma cells. J Mol Struct.

[6] Gong WJ, Pang YT, Wang CY, Wang WM, Wang HF (2025). Photoinduced NO release of [Fe2(µ-SL)2(NO)4] complexes and their protein adducts: insights from structure, cytotoxicity, and photodynamic studies. Inorg Chem Front.

[7] Timoshin AA, Vanin AF, Orlova TR (2007). Protein-bound dinitrosyl-iron complexes appearing in blood of rabbit added with a low-molecular dinitrosyl-iron complex: EPR studies. Nitric Oxide.

[8] Pokidova OV, Emel’yanova NS, Kormukhina AY, Novikova VO (2022). Albumin as a prospective carrier of nitrosyl iron complex with thiourea and thiosulfate ligands under aerobic conditions. Dalton Trans.

[9] Shumaev KB, Kosmachevskaya OV, Timoshin AA, Vanin AF, Topunov AF (2008). Dinitrosyl iron complexes bound with haemoglobin as markers of oxidative stress. Methods Enzymol.

[10] Shumaev KB, Kosmachevskaya OV, Nasybullina EI (2023). Antioxidant and antiradical properties of histidine-bound dinitrosyl iron complexes. Int J Mol Sci.

[11] Kosmachevskaya OV, Nasybullina EI, Shumaev KB, Novikova NN, Topunov AF (2020). Effect of iron–nitric oxide complexes on the reactivity of hemoglobin cysteines. Appl Biochem Microbiol.

[12] Wu CR, Huang YD, Hong YH (2021). Endogenous conjugation of biomimetic dinitrosyl iron complex with protein vehicles for oral delivery of nitric oxide to brain and activation of hippocampal neurogenesis. JACS Au.

